# Revealing the translator’s style: A corpus-based study of english translations of *Mencius*

**DOI:** 10.1371/journal.pone.0305894

**Published:** 2024-07-16

**Authors:** Yaoyao Gao, Guijun Zhou

**Affiliations:** School of Foreign Languages, Northeast Normal University, Changchun, China; University of Aizu, JAPAN

## Abstract

Based on the self-built English translation corpus of *Mencius*, this study conducts a lexical, syntactical and textual comparative analysis of *Mencius* English translations by James Legge (1861), Leonard A.Lyall (1932) and D.C.Lau (1970) through adopting a combination of quantitative and qualitative methods and employing Tokenizer, Tree Tagger, WordSmith8.0, AntConc and Readability Analyzer software. By analyzing representative translation examples and the para-text of each translation, this study explores the relationship among the historical background, translator’s cultural identity and translation motivation. The results reveal that the translator’s style is closely related to the translation strategy determined by the translation purpose rooted in translator’s cultural identity in different historical and social backgrounds.The study findings will bring a new perspective for the translator’s cultural identity research, contribute to the translator’s style study and deepen the understanding of the English translation and overseas dissemination of *Mencius* with the help of corpus technology.

## Introduction

*Mencius* is a collection of Mencius’ quotations compiled by his disciples and re-disciples during the Spring and Autumn Period, including Mencius’ political theory, philosophical system and ethical education thoughts. It is the Confucian classic with the most far-reaching ideological influence after *the Analects*, which plays a significant role in promoting development of Chinese culture and attracts plenty of scholars from all over the world to study and translate it. However, the English translation of *Mencius* is a unity composed of the translators, translation strategies, the process of translation and the interaction process among all the elements, and people must look at both the translated texts and their translators in order to achieve a better understanding of English translations of *Mencius*. In the process, the translator’s style has gradually attracted attention.

The concept of style has always been central to translation studies. In the early phases of translation studies, the view that translators need to make proper linguistic choices to reproduce the linguistic style and even the rhetorical elements of the source text in translated texts so that the target readers can experience the same effect when reading the translation as the source readers read the source text was dominant, which aroused the dissatisfaction of some scholars. They thought this approach regarded translation as a derivative and secondary activity and reinforced the dominant position of the source text. In challenging this, Bakery [1] proposed to use corpus method to study the style of the translator, and argued that translation is the translators’ unique and creative activities rather than a process of mechanically reproducing the style and meaning of the source text. However, with the deepening of the research, some researchers have realized that there is a basic conceptual problem that hinders the progress of the research, that is, the difference between the concepts of translator’s style and translation style. Hu and Xie [2] highlighted that “the translator’s style analyzed based on some translations is not the translator’s style in the real sense, but the translation style or translation language characteristics of the specific translation.” In the strict sense, the study of the translator’s style should select different translations by the same translator as the research object, and summarize the translation style based on analyzing the common characteristics of these translations in the use of language structure and the application of translation strategies and methods. Some scholars held different views about this. Huang [3] pointed out that the existing research on the translator’s style can be divided into two categories: The parallel model focuses on the processing of the same source text by different translators in the same target language, that is, “multiple translations of one text”, and specifically examines the regular processing ways of certain features in the source text in different translators’ translations [4–14], while the analogy model focuses on the difference between the whole of a translator’s translation and the whole of another translator’s similar translation by quantitatively examining language form parameters and objectively describing the personalized traces of the translator [15–17]. In this paper, we adopt the Huang’s definition of the translator’s style, and use the term “translation style” to refer to the unique language features that differentiate one translated text from other translated texts based on the same source text and into the same target language by multiple translators, that is, the translator’s style in parallel model.

Some scholars have used the corpus research method to study the translation style of Chinese classics. The focus of such research is generally on the distinct manner in which translators present the creation, including the choice of words, sentence structure, use of imagery and rhythm [18]. Zhao [19] discussed the translator’s style by studying the lexical density, relevant high frequency words and narrative perspectives of two English translations of *Tao De Jing*. Moreover, she found that different translation purposes lead to different translation styles. Wu Jingxiong paid attention to the acceptability of the translated text by Western readers; hence, the translated words are simple. Meanwhile, Wiley aimed to restore the accuracy of the translated text so that the lexical density is higher than that of Wu Jingxiong’s translation. Lv [20] studied the linguistic features at vocabulary (TTR and average word length) and sentence levels (ASL and standard deviation of sentence length) in two English translations of *The Analects of Confucius*, and identified that different translators adopted different translation strategies, tending more toward domestication (Gu Hongming) or foreignization (Arthur Wiley). Other studies on the translator’s style mainly focus on keywords [21], lexical richness [22] and discourse readability [23]. This line of research in corpus-based literary translation has significantly contributed to establishing the foundation by closely examining one or two specific aspects of style. However, style involves various possible features situated at different linguistic levels, not only features at the level of the sentence but also those that go beyond the sentence; thus, some researchers suggested that a greater variety of levels of language must be examined to uncover various aspects of style. Moreover, comparing the translator’s style only with the support of quantitative data is obviously superficial. We argue that, at least, the frequency of the discussed stylistic features should be combined with the para-texts of translations or relevant historical materials to explore the translator’s style to offer more powerful evidence for the translator’s study of literary translations, especially an immortal literary masterpiece such as *Mencius*. These limitations of the aforementioned research provide more space for the study of translator’s style in the future.

Based on historical records, the Western translation of *Mencius* began in the second half of the 16th century. David Collie completed the first English translation of *Mencius* in 1828. In addition to this version, translations by James Legge (1861) and Arthur Blackey Hutchinson (1882) completed in the 19th century were also published one after another. In the 20th century, several notable people translated *Mencius*, including Leonard A. Lyall (1932), W.A.C.H. Dobson (1970), Ch’u Chai and Winberg Chai (1965), and D.C.Lau (1970). Moreover, the first English translation completed by the Chinese scholar was published during this period. There have been 84 English translations of *Mencius* so far, of which 20 are complete reference translations.

With the emergence of many English translations of *Mencius*, studies on translations have emerged one after another. There are two main approaches to exploring style in translations of *Mencius*. Some scholars have focused on a particular translator of *Mencius* [24,25], whereas others have adopted comparative studies to examine multiple translations of *Mencius* [26,27]. In the following sections, we summarize the main studies reflecting the two approaches and identify potential limitations to provide the necessary context for our own study. Wu [24] analyzed the phenomenon of mistranslations in James Legge’s translation, and further emphasized that accurate understanding of the original text is the key to English translation of Chinese classics. Jin [25] discussed the concise style of David Hinton’s translation from three aspects, namely, the word choice, personal pronoun abbreviations, and English translation of titles. Hong [26] discussed the translation effect of argumentative speeches in the English translations of *Mencius* by James Legge (1861) and D.C.Lau (1970), and reviewed the translator’s practice. Wang [27] discussed the subjectivity of translators in the English translation of *Mencius* by James Legge (1861), Zhao Zhentao (1999), and Wu Guozhen (2015), which offered a new perspective for the study of English translations of Chinese classics. Although we have gained a basic understanding of the English translation study of *Mencius*, our review reveals that there are two major limitations to current research. Most existing studies rely heavily on subjective analysis of translated content to distinguish the translator’s style, which is not rigorous and scientific. Furthermore, a lack of stylistic comparison exists among multiple versions.

To fill the gaps in the above research, we construct a parallel corpus that contains three English translations of *Mencius* and analyze revelant subtext meterials and representative examples to identify the translator’s styles while analyzing their causes.

Therefore, we attempt to answer the following research questions:

(1) What are the linguistic features of three translations at the lexical, syntactic and discourse levels?

(2) What factors influence the formation of the translators’ styles?

Following these questions, The Research design section explains the materials and methods employed in this study. The Results and Reasons behind the translator’s style sections present the corpus analysis results of three English versions of *Mencius* and causes of different translators’ styles. The Discussion section discusses the findings of this study with relevance to previous related studies. The Conclusion section draws conclusions and highlights the limitations of the study with corresponding suggestions. The results of this study help readers to understand the differences between different translations and choose the translation suitable for their own reading and learning more easily, and also provide some suggestions for future translators to translate *Mencius*.

## Research design

### Materials selection

We choose the *Mencius* translated by James Legge (1861), Leonard A.Lyall (1932) and D.C.Lau (1970) as the corpus, and the collection of these translations follows the principles of representativeness and comparability. First, the three translators are from different historical periods and have different cultural identities, determining their respective translation motives, translation strategies and distinctive translations. Second, the three translations were translated from the same original text-the annotated version of *Mencius* by Zhuxi, indicating that these translations are highly comparable. Finally, prior to the corpus analysis, the author excludes the introduction, footnotes and other explanatory materials to ensure the thoroughness of the analysis.

### Research tools

This study employed corpus tools such as Tokenizer, Tree Tagger, WordSmith 8.0, AntConc and Readability Analyzer. Tokenizer is a software aimed at word segmentation. Tree Tagger using the Pennsylvania Tree Library code set is an automatic part of speech tagging software developed by Helmut Schmid at the University of Stuttgart, and it can automatically identify and label the part of speech of each word in English, German, French and Italian languages. WordSmith 8.0 is an integrated suite of programs developed by Mike Scott of Oxford University Press, including Wordlist, Keyword and Concord programs, which can produce a list of all the words, phrase or word clusters and examine how words behave in texts. Once the translated texts have been processed using WordSmith 8.0, the lexical features and syntactic features will be revealed. Moreover, AntConc is a tool that can be employed to search all kinds of words in the corpus, and Readability Analyzer can be utilized to count the passive sentence information and readability information of texts.

### Measurement

The specific linguistic features of the translated text mainly include lexical, syntactic and textual features [28].

The lexical features include the number of tokens, types, type/token ratio (TTR), standardized TTR (STTR), word length distribution, mean word length (MWL) and lexical density. The type/token ratio (TTR) reflects the richness of text vocabulary. Generally speaking, the higher the TTR is, the higher the vocabulary richness will be. Nevertheless, when the text length is long, the standardized type/token ratio (STTR) that refers to the type/token ratio every 1000 words is usually employed to compare the vocabulary richness among translated texts.

The word length distribution is the probability of the distribution of the word length (i.e., the number of letters that make up the word) in the text, and MWL refers to the average length of words, indicating the lexical complexity to some extent. WordSmith 8.0 can count the number of words of each length in a text and calculate the average word length. LD is the proportion of meaningful words in the entire text of a translation, which can reflect the amount of information contained in the text. It is an important indicator for analyzing the style of the translation. The higher the lexical density is, the greater the proportion of content words in the translation is, the greater the information carrying capacity is, and the relatively more accurate and complete the expression of the original meaning is.By contrast, the lower the lexical density is, the more functional words there may be in the translation, and the lower the informative ability of the text is. The content words in English usually include verbs, nouns, adjectives, adverbs, and numerals. It can be calculated by the following formula.

$$LD\;(lexical\;density)=\frac{total\;number\;of\;lexical\;word\;tokens}{total\;number\;of\;tokens}\times 100\%$$

The syntactic features include the number of sentences, average sentence length (ASL) and standard derivation of word numbers between sentences (STD). ASL and STD can reflect to some extent the complexity of syntactic structures, and are often considered as important makers to measure the language characteristics of the translated text and the translator’s style. Generally, the shorter the STD is, the simpler the syntactic structure of the text is, while the longer the STD is, the more complex the syntactic structure of the text is. The STD can be calculated by the following formula.

$$STD=\frac{the\;average\;sentence\;length}{the\;total\;sentence\;length}$$

The textual features are the overall expression of the translator’s style, which can be described from numerous aspects. The most important indicators are cohesion and readability information. Cohesion is a fundamental attribute and important feature of one discourse, reflecting the overall and hierarchical sense of the discourse structure. As an important cohesive device, conjunctions enable various logical relationships between clauses in the discourse, helping readers analyze the semantic relationships between sentences and more effectively understand the text. In this study, we manually annotate various conjunctions in the text, use AntConc software to search them individually in each translation, summarize different types of conjunctions, and subsequently calculate their numbers. Text readability refers to the difficulty of understanding a text, which can be employed to test whether the translation is easy for readers to understand at a macro level. The Readability Analyzer can detect the difficulty of reading a text by calculating the Flesch Readability Index of one text. The calculation formula of Flesch Reading Index is as follows:

FleschReadingIndex=206.835‐(1.015*ASL)‐(84.6*ASW)

(ASW refers to the average monosyllable numbers of words. The higher the Flesch Reading Index is, the less difficult the text is.)

### Procedure

After determining the English texts and corpus software needed for the research, we began to clean the corpus, segment the text words and tag the parts of speech of texts.

#### Corpus description

We download the PDF versions directly from the Internet and convert them directly to TXT format. To ensure the quality of the corpus and the reliability of the research, we manually remove redundant spaces and misspelled and garbled characters from the corpus, and save it as James Legg’s *Mencius*.txt (46,966 tokens), D.C.Lau’s *Mencius*.txt (52,675 tokens) and Leonard’s *Mencius*.txt (45,780 tokens).

#### Word segmentation and corpus tagging

First, we import the processed corpus into the Tokenizer application, click the convert button to obtain the segmented file and save the files as Jame Legg’s *Mencius* 2.txt, D.C.Lau’s *Mencius* 2.txt, and Leonard’s *Mencius* 2.txt respectively.

Second, we open the WordSmith8.0 software; enter the WordList interface; click the File button; select Jame Legge’s *Mencius* 2.txt, D.C.Lau’s *Mencius* 2.txt, and Leonard’s *Mencius* 2.txt files; and click “make a word list” button to enter the statistics interface. Thus, we can obtain the vocabulary and sentence information of the three translations as Table 1, 2 and 4.

**Table 1:** Lexical statistics features of the three translations of *Mencius*.

TT	Tokens	Types	TTR	STTR
Legge	46,966	4,144	8.82	37.92
Leonard	45,780	3,671	8.02	34.53
D.C.Lau	52,675	4,262	8.09	35.83

**Table 2:** Main distribution statistics of word length in the three translated versions of *Mencius*.

Word Length Translations	Legge	Leonard	D.C.Lau
MWL	4.38	4.02	4.08
1-letter words	4.47%	4.49%	3.35%
2-letters words	21.2%	15.3%	16.2%
3-letters words	24%	25.3%	21.7%
4-letters words	18.3%	20.2%	19.8%
5-letters words	10%	15%	12.1%
6-letters words	10.4%	9.83%	11.8%
7-letters words	6.32%	5.76%	8.38%
>8-letters words	9.11%	6.42%	8.42%

Third, we open the Tree Tagger for Windows 3.0 software; click the file; open the File button in the upper left corner; select the three files; set the language to English, the encoding to ANSI, and output form to Word_POS; and then click the Run Tagger button to get the TAGGER files. Subsequently, we name the files James Legge’s *Mencius* TAGGER.txt, D.C.Lau’s *Mencius* TAGGER.txt, and Leonard’s *Mencius* TAGGER.txt.

Fourth, based on the part of the speech coding table of Tree Tagger, AntConc’s Concordance retrieval function is used to search the number of content words, as shown in Table 3.

**Table 3:** Tagging statistics and LD of the three translations of *Mencius*.

TT	Noun	Verb	Adj.	Adv.	Content Words	LD (%)
Legge	11,609	9,915	2,962	1,814	26,300	56
Leonard	13,073	8,752	1,573	865	24,263	53
D.C.Lau	14,398	10,412	3,457	2,747	31,014	59

Fifth, we manually annotate and count the conjunctions in the documents, as shown in Table 5.

Sixth, we use the Readability Analyzer software to obtain readability information about texts, as shown in Table 6.

## Results

### Lexical features

Tables 1 and 2 display the lexical statistics produced by WordSmith Wordlist.

Based on the data results, D.C.Lau’s translation of *Mencius* contains the most tokens and types, followed by Legge and Leonard’s translations. All three translations are longer than the original text of *Mencius* containing 36,970 tokens, which indicates the three translations tend to highlight the implicit information present in the ancient text, making readers comprehend the text more easily. Regarding the STTR analysis, it was found that the STTR of Legge’s translation is 37.92, higher than 35.83 that of D.C.Lau’s translation, showing that Legge adopts a more diverse lexicon in his translation. The STTR of Leonard’s translation is 34.53, showing Leonard uses the narrowest vocabulary. There is a longer MWL and a higher percentage of words longer than eight letters than those of Leonard’s version in D.C.Lau’s translation, suggesting that the words are longer and relatively more complex in D.C.Lau’s translation.

The author calculates the number of content words in the three translated texts of *Mencius*, and Table 3 presents the results.

The LD of the D.C.Lau, Legge and Leonard’s translations are 59%, 56% and 53% respectively, all of which are over 50%. Among them, the LD of D.C.Lau’s translation is the highest, followed by that of Legge and Leonard’s translation. The result of Laviosa’s research on the LD of the English source language corpus and the target language corpus shows that the LD of the former is 54.95% and the latter is 52.87% [28]. The LD of the three translations is higher than that of the target language, especially that of D.C.Lau’s translation far exceeds that of the source language. Thus, it can be concluded that D.C.Lau’s translation has the largest amount of information compared with the other two translations. There is the lowest lexical density in Leonard’s translation, which indicates that Leonard chooses to add functional words to simplify the translation and make the translation structure more clear and easy to understand.

### Syntactic features

The WordSmith Wordlist Program is also used to calculate the number of sentences, average word numbers per sentence and standard deviation of word numbers between sentences, revealing the syntactic features of each translation. The statistics are shown in Table 4.

**Table 4:** Data features of three translations at sentence level.

	Legge	Leonard	D.C.Lau
Sentence Number	3261	2985	3052
Mean (in words)	18.42	17.26	17.32
STD	13.71	10.42	11.54

According to the statistical data of WordSmith 8.0, the result shows that the average sentence length of translations by James Legge, D.C.Lau and Leonard are 18.42, 17.32 and 17.26 respectively, indicating that the average sentence length of Legge’s translation is the longest, and that of Leonard’s translation is the shortest. From the perspective of language, there are many short sentences in the original text of *Mencius* and the style is concise. Leonard uses short sentences to produce his translation, which reflects the characteristics of the source language. The average sentence length of Legge and D.C.Lau’s translations are relatively longer, probably because the two translators emphasize the expression of information and transform the conversational language of the original text into narrative language. The STD of Legge’s translation is 13.71, higher than that of the other two translations, which indicates that the sentence length structure is more diversified.

### Textual features

We have conducted a statistical analysis of the usage of conjunctions in each translation and compared the overall readability among three translations. The results are shown in Table 5 and 6.

**Table 5:** The use of conjunctions in three translations.

	Legge	Leonard	D.C.Lau
Coordinate Conjunction	150	138	218
Causal Conjunction	104	115	117
Adversative	112	109	138
Temporal Conjunction	106	137	147
Conditional Conjunction	46	39	53
Total	518	402	673
Total Vocabulary	27304	25405	29801
Frequency (%)	1.74	2.31	2.46

**Table 6:** Overall readability of three translations.

	Legge	Leonard	D.C.Lau
Overall Readability	73.81	79.81	76.13

Table 5 reveals that there are certain similarities and differences in the use of conjunctions in the three translations. First, both translators can clearly convey the various semantic relationships implied in the original text to the reader by adding various conjunctions (e.g.,coordinate conjunction, causal conjunction, temporal conjunction, and conditional conjunction) in the translation, which is consistent with the finding that the tokens of three translations are greater than that of the original text. However, we can also clearly see that D.C.Lau used a higher percentage of conjunctions in translation compared to Leonard and Legge, indicating that he tended to simplify linguistic differences between the original text and the translated text to make the translation easier for readers to understand. It is the use of more conjunctions that results in the longer average sentence length of Lau’s translation.

Simultaneously, we can find that the readability of Leonard, D.C.Lau and Legge’s translations are 79.81, 76.13 and 73.81 respectively, indicating that Legge’s translation is the most difficult to understand and this may be related to the diversified vocabulary, complex sentence structure and fewest conjunctions in Legge’s translation. The readability of Leonard’s translation is the highest, indicating Leonard’s translation is the easiest to read, which is consistent with his translation’s characteristics of the simplest words and sentence structure and the most conjunctions.

In summary, we can see that Legge’s translation has the highest STTR, longest MWL and highest lexical density, indicating the vocabulary is richer and more diverse and his version has the longest mean sentence length with the highest standard deviation, pointing to a complex syntactic structure. Conversely, he uses the fewest conjunctions, making his translation relatively challenging to read. Leonard’s translation has the least varied vocabulary and the shortest MWL with the shortest mean sentence length, making the sentence structure simple. D.C.Lau’s translation has the highest lexical density, indicating a sizable information load and a higher mean sentence length with a greater standard deviation, suggesting a relatively complicated and variable syntactic structure. Meanwhile, the higher percentage of conjunctions and the highest readability of Leonard’s translation indicates that the text is easiest to understand. We can see the following example obtained from the translations.

ST1: 若杀其父兄, 系累其子弟, 毁其宗庙, 迁其重器, 如之何其可也?

[29]

TT1: But you have slain their fathers and elder brothers, and put their sons and younger brothers in confinement. You have pulled down the ancestral temple of the State, and are removing to Ch’i its precious vessels.

[30]

TT2: Can it be right to kill their fathers and elder brothers, bind their young men, pull down their ancestral temples, and carry off their heavy ware?

[31]

TT3: How can it be right for you to kill the old and bind the young, destroy the ancestral temples and appropriate the valuable vessels.

[32]

The use of separate verbs in English is usually on a more formal occasion.There are many dialogs between Mencius and various princes in *Mencius*, which is a type of dialog genre with a kind of relatively colloquial style. When translating such verbs, using phrasal verbs is reasonable. Respecting the colloquial language characteristics of the original text, James Legge uses two separate verbs (slain and removing) and two phrasal verbs (put… in confinement and pull down) to explain the original text, suggesting the richness and diversity of vocabulary. The sentence structure of Legge’s translation comprises three compound sentences, while there is only one interrogative sentence in D.C.Lau and Leonard’s translations, indicating the complexity of sentence structure in Legge’s translation. D.C.Lau uses a series of single verbs “kill, bind, destroy and appropriate” to translate the single verb in the original text, turning the positive sentence pattern of the original text into a special question guided by the word “how”. It accurately conveys the meaning of the original text and enhances the translator’s condemnation to those immoral behaviors. Leonard, conversely, uses simple and phrasal verbs to convey the meaning of the original text, making the sentence structure simplest and the translation easiest to read.

## Reasons behind the translator’s style

Baker [1] once said that “identifying the translator’s language habits is not the purpose of studying the translator’s style, and its value lies in that it can tell us the cultural and ideological position of a translator or most translators or it can tell us the cognitive process and mechanism that affect the translation behavior”, which shows the necessity and significance of the analysis of the causes of the translator’s style. Hu Kaibao and Xie Lixin [2] believed that the causes of the translator’s style include the translator’s own factors, which involve the translator’s identity and his/her translation concept (e.g.,translation motivation, purpose, strategy and method), and non-translator factors, which primarily refer to the historical period and social and cultural contexts. The next section will discuss the reasons why translators form different styles based on their cultural identity and translation motivation in specific historical and sociocultural contexts through the analysis of representative examples and para-textual of translations.

### The missionary in the 19th century

In the early 19th century, a large number of missionaries entered China to study Chinese culture in order to smoothly preach. James Legge (1815–1897) was a missionary of the London Missionary Society in the 19th century. In 1838, he entered the University of London to study Chinese and arrived in Hong Kong, where he served as director of Anglo-Chinese College and devoted himself to spreading Christianity and learning Chinese. After constant contact with the Chinese people and extensive reading of Chinese classics, Legge was deeply impressed by the profound ancient Chinese culture and began to translate *The Chinese Classics*, which was successfully published in 1861 [33]. In the preface, he wrote the following:

I look at Chinese philosophic classics and am eager to learn about its language, history, literature, ethics and social formation. It seems to me that a Westerner, especially a missionary, can not be qualified to assume the responsibilities required by his present position as a missionary until he has mastered the Chinese classics and examined all the fields of thought of the ancient Chinese sages [30].

The purpose of Legge’s translation work is to open the door to Chinese culture for missionaries and serve missionary work based on deepening the understanding of traditional Chinese culture. In 1893, in the first volume of the revised edition of *The Chinese Classics*, we find Legge’s high praise for *Confucius* and *Mencius*, he once said the following:

I hope I have not treated them unfairly, and the more I study their characters and opinions, the more I respect them. Their influence on the Chinese people is great and beneficial, and their teachings are also important lessons for those of us who profess to be Christian so that faithfulness to the original Chinese classics is the first principle. Not that I am indifferent to the value of an elegant and idiomatic rendering in the language of the translation, and I hope that I was able to combine in a considerable degree correctness of interpretation and acceptableness of style [34].

Therefore, “faithfulness” is the first principle of Legge’s translation of the classics. He used the foreignization translation strategy and the literal translation method to complete his translation. He not only pursued the formal consistency between the translation and the original text but also used extensive and detailed annotations as well as indexes to further express his understanding of the work. In his translation, he deliberately broke target conventions by retaining something of foreignness of the original, hence, the translation has the characteristics of diversified vocabularies, complex sentence structures and low readability.

We can see the following example extracted from the *Mencius* translation by Legge:

ST2: 孟子曰:地方百里而可以王

[29]

TT4: Mencius replied, ‘With a territory which is only a hundred li square, it is possible to attain to the royal dignity.’

[30]

TT5: Mencius said, A land of a hundred square miles may rule the kingdom.

[31]

TT6: ‘A territory of a hundred li square,’ answered Mencius, is sufficient to enable its ruler to become a true King.

[32]

From the perspective of sentence pattern, Legge completed the translation with two clauses, and the sentence structure is the longest. Translators have different translations of “里(li)” that is a unique Chinese unit of measurement. Legge and D.C.Lau translated “里” as “li”, and they explained the word “li” in their notes, accurately conveying the connotation of the Chinese unit. Leonard chose to translate “里” using the word “mile”, which is familiar to foreigners. The advantage of this translation is that it is convenient for foreigners to understand; However, it is easy to westernize the unique Chinese cultural words, which causes unnecessary association, and is not conducive to the spread of traditional Chinese culture.

James Legge devoted his whole life to the translation and interpretation of Chinese classics, realized the transformation from a missionary to a sinologist and opened up the history of the spread and acceptance of traditional Chinese Confucianism in the West with Chinese classics as the carrier. Therefore, his translation is widely regarded as the authoritative translation.

### The customs officer in the first half of the 20th century

Zhan [35] pointed out: “From the middle of the 19th century to the first half of the 20th century, foreigners working in the Chinese Customs gradually realized that despite of different cultural backgrounds, they must be familiar with the Chinese language, which was an important step for the customs to gain a firm foothold and expand its foundation, so they consciously or unconsciously acted as a bridge to introduce the East to the West and opened the curtain of ancient China for the Western world. It makes the Western world feel the charm of ancient Chinese civilization, and promotes the development of international Sinology research.”

Leonard A.Lyall (1867–1934) is a customs officer who has worked in the Chinese customs for 40 years and made various achievements in translating ancient Chinese classics. In his spare time, he translated the Confucian classics *The Analects of Confucius* (1909), *The Mean* (1927), and *Mencius* (1932) into English, and assisted the German Karl E. G. Hemeling in compiling the *English-Chinese Dictionary* to facilitate foreigners to get familiar with and understand the traditional Chinese characters [36]. In the preface to the English translation of *Mencius*, Leonard A.Lyall said the following:

I have tried to make my translation as nearly as possible a word-for -word rending of the Chinese to reappear the simple style of *Mencius*. Where I have failed, I have usually given the literal translation in the notes.

The most important Chinese words have always been translated by the same English words;e.g. jen, love; chun-tzu, gentleman; tao, the Way; yi, right, or once or twice justice. The only exception is li, which I have sometimes translated in good form, sometimes courtesy, and sometimes ceremony [31].

Leonard’s translation can be observed to be primarily literal, emphasizing the consistency of word meaning and sentence structure with the original text and striving to present the concise style of the translation with simple and vivid words and short colloquial sentences; therefore, the translation has a strong readability. However, he overemphasized the literal meaning of words and did not accurately convey the connotation of the original text to some extent. In order to reduce the cultural strangeness of Western readers and draw them closer to the original work, Leonard adopted the domestication translation strategy. That is, he attempted to find similar words familiar to Westerners in English to translate unique cultural words, ignoring the unique meanings of words in specific contexts and reducing the fidelity of the translation to the original work.

ST3:《诗》云“昼尔于茅, 宵尔索綯; 亟其乘屋, 其始播百谷”

[29]

TT7: In the day-light go and gather the grass,

And at night twist your ropes;

Then get up quickly on the roofs;

Soon must we begin sowing again the grain.

[30]

TT8: Out in the reeds by day;

Binding, plaiting by night;

Climb the roof betimes;

There are hundreds of seeds yet to sow.

[31]

TT9: In the day time they go for grass;

At night they make it into ropes;

They hasten to repair the roof;

Then they begin sowing the crops; [32]

Leonard used simple words and short sentence patterns to explain the original text and retained the concise style of the original text. The biggest difference among three translations is that the translation of “亟其乘屋 (ji qi cheng wu),” both Legge and Leonard took the original meaning of “乘(cheng)” and translated it as “quickly climbing on the roof.” Although the translation can reflect the literal meaning of the original, it broke the relationship between the verses and was not easy for readers to understand. However, D.C.Lau took the extended meaning of “乘(cheng)” and translated it into “hurry to repair the roof” which is more accurate and understandable.

In contrast to Legge’s extensive footnotes, there are only a few brief notes in Leonard’s translation. In the preface of *The Analects of Confucius*, he explained the following:

Since I am addressing the general English audience, too long an extraneous note will only look out of place [31].

Although there are few annotations, the most detailed and clear catalog is shown in Leonard’s translation. This translation includes one table of contents, readers can easily find their own interest in the content pages just by flipping it. Leonard A.Lyall always puts the acceptance of ordinary readers in the first place, therefore, his translation is more popular and widely welcomed by ordinary readers.

### The devoted scholar in the second half of the 20th century

In the middle and late 20th centuries with the transfer of Western sinology centers to the United States, American increased its investment in Sinology research, and the English translations of Chinese philosophical classics published have a wide range of coverage and influence. During this period, Chinese scholars in the United States became an important force in the English translation of Chinese philosophical classics, because Chinese translators had a more accurate and profound understanding of Chinese classics and were able to gradually present the philosophical connotation and cultural essence of Chinese classics to American readers [37].

D.C.Lau (Liu Dianjue,1921–2010) is a famous sinologist, translator and philosopher. In 1950, he graduated from the Glasgow University and taught at the London University. During his 30 years of teaching, he has devoted himself to the study of Chinese classics as well as translation work. In the 1960s and 1970s, D.C.Lau translated *Tao Te Ching* (1963), *Mencius* (1970) *and The Analects of Confucius* (1979) for the Penguin Classics series. He once recalled the origin of translation:

After I twice refused with the publication of other people’s translations of *Tao Te Ching*, the editor was a little unconvinced, and he wrote to me and said, “Since you said that they can’t translate it, why don’t you try it yourself?” This is why I translated *Tao Te Ching,* At that time, the publishing industry was flourishing, they wanted to develop the section of Chinese classics, and asked me which books could be translated, I suggested *Mencius*, and this time it took me seven years to translate the book… ..[32].

To not misinterpret Lao Tzu’s philosophical thoughts, D.C.Lau decided to translate Chinese classics by himself with a rigorous and serious attitude after refusing the publication of two translations. Later, D. C. Lau translated *Mencius*. His main purpose was to introduce the essence of Chinese philosophy to the readers in the target language world more accurately and completely. Therefore, he adopted the foreignization translation strategy and the “thick translation” method and used a variety of auxiliary text materials to interpret the translation. Deng [38] once commented on the translation of D.C.Lau’s *Mencius*:

Professor Liu has the academic foundation of Qianjia period since childhood, and especially loves the exegetic knowledge of the Er Wang. Coupled with the modern Western academic training, he is not limited to knowledge of one school, his insights and propositions held have a certain basis, and the harvest of classic research can be said to be beyond the scholars of Qianjia period. The translation is rooted in deep learning, and there is deep insights in the judgement of every word and sentence [38].

To some extent, scholars’ high evaluation of D.C.Lau’s translation reflected the features of rigorous and accurate words in the translation. However, Chen [39] criticized the *Lao Tzu* translation for not specifying annotations or other documents supporting his translation. In response to this criticism, Liu responded as follows:

The target audience of my translation is mainly the general public readers, so there is no need to trace the source in the annotation, otherwise it can only arouse the interest of sinologists in the West, not the general public. And I translated Chinese Classics at the request of Penguin Publishing, which is one of the largest publishers of popular books in the world, and its “Penguin Classics” series is characterized by common readers’ popularity [32].

His translation showed the simple style of *Mencius* and expressed his profound thoughts. D.C.Lau has a strong reader awareness. Although there are various notes in his translation, he does not excessively present too much explanation and rationale and leaves the reader a broader space for thinking. Moreover, to eliminate the barriers between Chinese and English, and help readers understand the content outside the text, the translation contained 40 pages of introduction and six appendices. In the introduction, Mencius’ life and thoughts are emphatically introduced, and the connotation of some important thoughts is explained in detail. His translation strategy is conducive to the wide dissemination of the translation in the West. This will greatly help Western readers living in different cultural backgrounds to understand Mencius’ thoughts from all angles. For his translation,Yang [40] thinks that “there are short sentences, concise and symmetrical structure in D.C.Lau’s translation, which reflects the oral dialog characteristics of the original text and is easy to understand.”

## Discussion

The Irish translation theorist Michael Cronin [41] once said, “If ideology was the main factor influencing cultural communication, now cultural identity has become the main mode of communication”. American translation theorist Edwin Gentzler [42] also emphasized the importance of cultural identity in his book *Translation and Cultural Identity*. He pointed out that the translator’s duty is to promote the communication and understanding of different cultures, so the translator is a cultural intermediary based on nationality, and the national identity is the most important cultural identity of the translator, which subtly affects the translator’s translation motivation and the choice of translation strategy, and thus determines the translator’s translation style. The translator’s cultural identity is closely related to translation activities.

James Legge’s serious and earnest attitude in translating Confucian classics won him high praise from Chinese and the western scholars. He believes that Christian thought and Confucianism are not absolutely opposed, and they can absorb nutrients from each other. The more a person possesses the spirit of Christ, the more he will view other religions with enthusiasm and fairness, meanwhile viewing his own beliefs without bias [43]. In terms of the principle of translation, Legge posits that the authority of the classics dictates that “faithfulness” must be the paramount translation principle. He adopted the foreignization translation strategy and the literal translation method to pursue the formal equivalence between the translated text and the original text, and added a large number of annotations in the translated text so that Western readers can appreciate the practical educational role of Chinese classics.

Although Legge made the translation incoherent to maintain the true meaning of the original text, we must admit his true-seeking attitude as a scholar and his infinite respect and tolerance for Chinese culture based on the Christian cultural identity.

The connection between Legge’s cultural identity and his translation activity can also be seen in other sinologists with western cultural identity. Their western cultural identity is the same, and there is no obvious difference in their desire to seek the truth of Chinese culture in the translation of Confucian classics. The difference is only the missionary or sinologist identity assigned by the times. Leonard once said: “Chinese culture is the most worthwhile culture to explore. *The Four Books* has more influence on the Chinese, and is an important way to understand the Chinese [44].” The main purpose of his translation is to help ordinary Western readers understand the cultural characteristics of the Chinese people. He adopted the domestication translation strategy and the free translation method, flexibly used subject-subordinate sentence patterns to make the translated text present explicit features and made use of extensive connectives and pronouns to increase the coherence and logic of the text to make the translation easier for Western readers to understand. Despite their different cultural identities, Legge and Leonard’s statements [45] about the motives and goals of translation clearly showed the same cultural position. “What we need to do is not just study Chinese traditions, but try to turn them into a cultural resource that enriches and transforms our own world.” This statement indicated that their motivation to study and translate Confucian classics is to serve the development of their own culture, while the so-called acquisition of true knowledge is only a yearning to achieve their behavioral goals, and the ultimate goal is to enrich and transform Western culture. In other words, although the translation strategies of Western missionaries and Sinologists are different, either domestication or foreignization, there is no difference in the goal orientation of their translation behavior that is to interpret and translate for the construction of Western culture, and these are determined by their Christian cultural identity.

The translation of *Mencius* by the Western missionaries and sinologists represented by James Legge and Leonard showed their Western cultural identity, while D.C.Lau’s translation of *Mencius* showed his Chinese cultural identity. In the preface of the translation of *Mencius*, D.C.Lau [32] proclaimed his recognition of Chinese culture. He said: “If the criterion of civilization is whether the rich can enjoy themselves, the Chinese civilization has undoubtedly failed. But if civilization is judged by whether people have a strong and binding sense of moral responsibility, then I can demonstrate that Chinese civilization has not failed even today. On the contrary, it was very successful.” This not only reflected D.C.Lau’s deep understanding of the essence of Chinese culture but also presented his full recognition of Chinese culture. Due to his special educational experience, D.C.Lau did not stick to the form of the original text but adopted the foreignization translation strategy and the thick translation method. He used rigorous and standardized long sentences and a large number of substantive words to retain the cultural meaning of the original text and added conjunctions and philosophical explanations to the original material in many places, which reflected the translator’s deep understanding of Chinese culture. Lin Yutang [46] once commented on D.C.Lau’s translation: D.C.Lau’s special educational experience made his translation come from the mastery of two languages and the understanding of their profound meanings, and the translator acted as a plating maker between Eastern and Western concepts. Through the injection of philosophical thoughts, the translator achieved the precise match of expression methods and meanings in his translation, which is a real revelation. It can be said that D.C.Lau’s translation activities at that time, as a specific individual’s activity under a specific era, realized the mutual integration and cooperation of two languages and cultures. His translation of *Mencius* and its expression of Chinese cultural identity are the results of many factors’ interaction including the specific historical stage, Chinese traditional culture, and his cultural position.

The English translation of Chinese classics is a kind of social and cultural practice in a specific historical stage. Different historical backgrounds give translators different cultural identities and determine the corresponding translation goals and translation strategies. Based on this understanding, there is no need to be entangled in the dilemma of the source language or the target language’s cultural orientation, and it is also one-sided to discuss the gain or loss of the translation strategy and translation goal without historical dimension considering. In the current study, the use of corpus-based research methods enables us to report the influence of these factors on translator’s style in a more objective and balanced manner.

## Conclusion

This study employs corpus technology to examine the stylistic patterns of three well-known English translations of the acclaimed Chinese classic *Mencius*, reveals the inner relationship among the historical and cultural reality, the translator’s cultural identity, the translator’s motivation and the translator’s style, and brings a new perspective for the study of the translator’s cultural identity in the field of translation studies. It is found that the missionaries in the 19th century adopted foreignization translation strategy and literal translation method to preserve the linguistic features and connotations of the original texts, which enhanced their understanding of Chinese culture and promoted their missionary work. At the same time, they express their own views with the introduction, which becomes the main supporting material for exploring the translator’s style of the missionaries; The first half of the 20th century was a turbulent time for the Western world, western translators often used demonstrating translation strategy and literal translation method to interpret the original text to facilitate foreign readers to understand the profound Chinese culture, but there were also cases of mistranslations, and during this period, the translated text became the main research material of translation style; In the 21st century, translators pay more attention to the deep connotation of the original text, and the translator uses the “thick translation” translation method to interpret the translation while using a variety of auxiliary text materials to explain the text to obtain the true connotation of the text, and the translator’s style can be found in both the text and the auxiliary text. However, it should be pointed out that this study has the following shortcomings: First, the corpus size is relatively small, and the language features in the study only represent a part of the translator’s style. In the future, abridged translations, compilation versions and a wider range of language features should be included in the research scope to expand the corpus size and obtain more comprehensive translator’s style characteristics. Second, the research method and perspectives are relatively traditional, necessitating that its level and angle of description be further broadened. In the following study, we can explore the transformation of the narrative stylistic features of *Mencius*’s source text in the translation from the perspective of narratology so as to update the method and broaden the level and depth of the research. We hope that this preliminary study will be the first step toward increased corpus-based translator’s style studies using parallel models in the future. In subsequent studies, these limitations must be supplemented to expand the existing research base of corpus-based stylistic studies and literary translation studies so as to conduct further research in this field.

## Supporting information

S1 File The quantitative data for the three translations. (XLSX)
